# Human Brain Project Partnering Projects Meeting: Status Quo and Outlook

**DOI:** 10.1523/ENEURO.0091-23.2023

**Published:** 2023-09-04

**Authors:** Angeliki Lorents, Marie-Elisabeth Colin, Ingvild Elise Bjerke, Simon Nougaret, Luca Montelisciani, Marissa Diaz, Paul Verschure, Julien Vezoli

**Affiliations:** 1 EBRAINS AISBL, Brussels B-1170, Belgium; 2 Neural Systems Laboratory, Institute of Basic Medical Sciences, University of Oslo, Oslo 0372, Norway; 3 Institut de Neurosciences de la Timone, Unité Mixte de Recherche 7289, Aix Marseille Université, Centre National de la Recherche Scientifique, Marseille 13005, France; 4 Cognitive and Systems Neuroscience Group, Swammerdam Institute for Life Sciences, University of Amsterdam, Amsterdam 1098XH, The Netherlands; 5 Institute for Advanced Simulation (IAS), Jülich Supercomputing Centre (JSC), Forschungszentrum Jülich GmbH, Jülich 52428, Germany; 6 Donders Center for Neuroscience (DCN-FNWI), Radboud University, Nijmegen 6500HD, The Netherlands; 7 Ernst Strügmann Institute (ESI) for Neuroscience in Cooperation with Max Planck Society, Frankfurt am Main 60528, Germany; 8 Institut National de la Santé et de la Recherche Médicale Unité 1208, Stem Cell and Brain Research Institute, Université Claude Bernard Lyon 1, Bron 69500, France

**Keywords:** applied brain models’ brain circuits and simulations, EBRAINS, HBP Partnering Projects, Human Brain Project, primate brain specifics

## Abstract

As the European Flagship Human Brain Project (HBP) ends in September 2023, a meeting dedicated to the Partnering Projects (PPs), a collective of independent research groups that partnered with the HBP, was held on September 4–7, 2022. The purpose of this meeting was to allow these groups to present their results, reflect on their collaboration with the HBP and discuss future interactions with the European Research Infrastructure (RI) EBRAINS that has emerged from the HBP. In this report, we share the tour-de-force that the Partnering Projects that were present in the meeting have made in furthering knowledge concerning various aspects of Brain Research with the HBP. We describe briefly major achievements of the HBP Partnering Projects in terms of a systems-level understanding of the functional architecture of the brain and its possible emulation in artificial systems. We then recapitulate open discussions with EBRAINS representatives about the evolution of EBRAINS as a sustainable Research Infrastructure for the Partnering Projects after the HBP, and also for the wider scientific community.

## Visual Abstract

**Figure:**
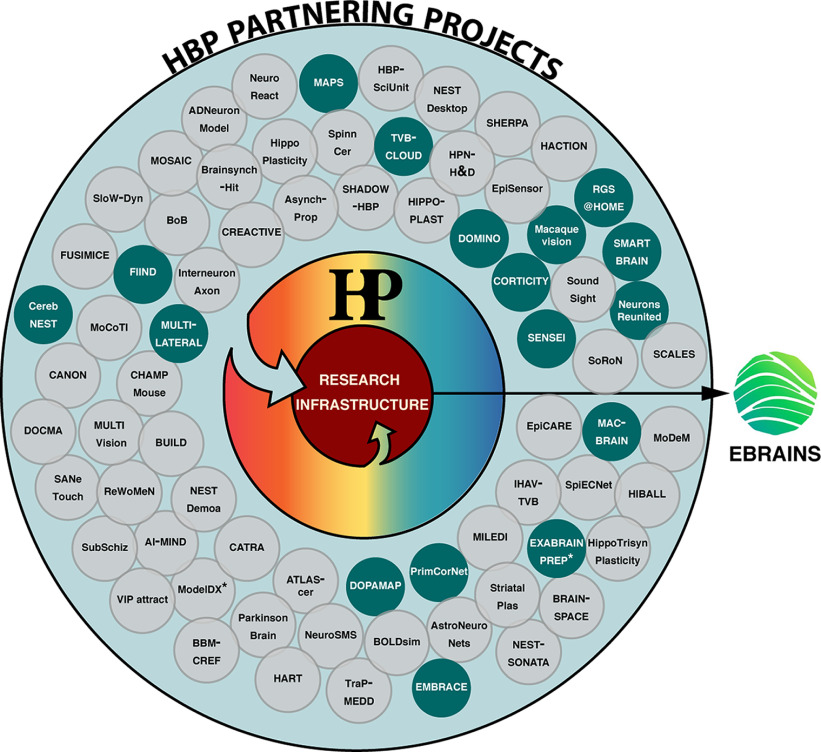

## Significance Statement

The Human Brain Project (HBP) is a 10-year European large-scale scientific project that aims to simulate functions and structure of the human brain, bringing together several fields of neuroscience from fundamental to clinical research to develop new therapeutic strategies for the treatment of neurologic diseases. The HBP Partnering Projects (PPs) program has been one of the measures to open up the HBP and promote sharing of resources and knowledge among researchers. We report here some of the achievements made by the HBP Partnering Projects that participated in a dedicated workshop in September 2022 and provide future prospects of this program in regard to the EBRAINS Research Infrastructure (RI).

## Introduction

### The Human Brain Project and the Partnering Projects

The Human Brain Project (HBP) is one of the two Future and Emerging Technology (FET) European-Funded Flagship projects launched in 2013, the second being the Graphene Flagship. It is one of the largest European research projects, with >500 scientists and engineers at over than 120 institutions across Europe coming together to address brain research targets and support the development of the digital distributed Research Infrastructure (RI) called EBRAINS (Human Brain Project, 2023).

**Figure 1.**
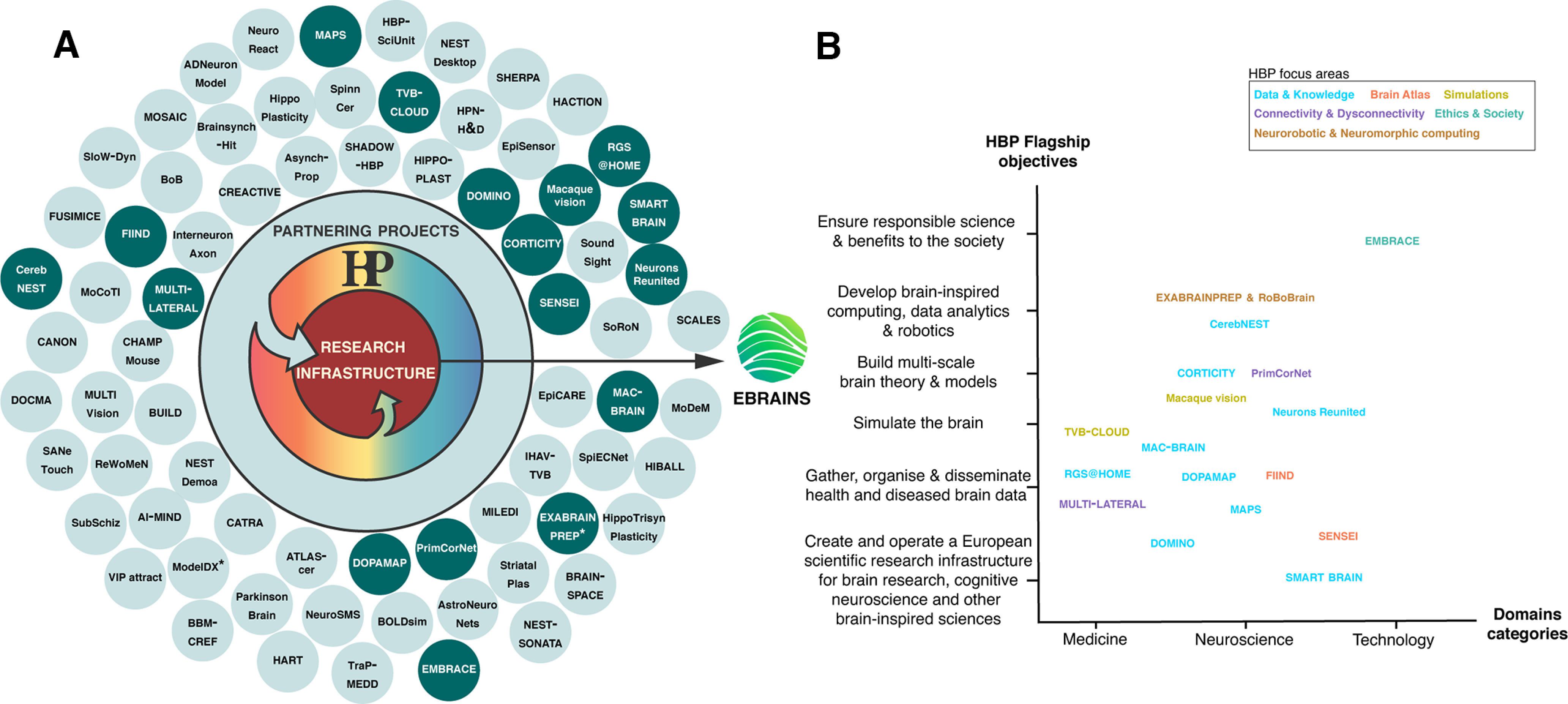
HBP Partnering Projects and relation with the HBP Flagship objectives. ***A***, Partnering Projects (PPs) have taken a complementary role to the HBP Core projects (rainbow color) in using and developing new tools through the HBP research infrastructure services which have evolved to EBRAINS. All partnering projects in clear green; projects present in HBP PPs Meeting in dark green. Note: Projects are ordered chronologically, from the earliest projects starting at 9 o’clock to the latest clockwise. ***B***, PPs classified in domain categories based on the field of research objectives and matched with HBP Flagship objectives. For clarity, only PPs that presented during the HBP PPs Meeting are included in this figure.

The HBP aims to achieve the so-called HBP Flagship objectives listed in Figure 1*B* (see HBP Framework Partnership Agreement: HBP FPA).

Both Flagship initiatives developed and implemented a partnering mechanism. The aim of the HBP Partnering mechanism was to allow already funded scientific projects to add new knowledge, competencies, ideas, and resources to the HBP and to benefit from the Research Infrastructure and the other capabilities made available by the HBP (see HBP FPA). Therefore, the activities of the Partnering Projects (PPs) were important to the work conducted by the HBP Consortium for the fulfillment of the aforementioned HBP objectives.

To ensure complementarity with the HBP scientific roadmap, Partnering Projects’ (PPs’) applications received were reviewed by HBP experts and then approved or rejected by the Science and Infrastructure Board (SIB) of the HBP. The SIB provides scientific leadership to the HBP, drives forward the HBP’s scientific excellence, ensures the implementation of its research plan, and develops the Project’s long-term scientific roadmap. Over the lifespan of the HBP, 76 PPs have emerged (Fig. 1*A*), which were composed of 92 HBP Consortium partners and 115 associated members (namely, partners that are not part of the HBP Consortium) and were categorized based on their research objectives in three main areas: medicine, neuroscience, and technology.

Each PP had a distinct research focus and their funding sources varied. Some of these projects were funded by a dedicated stream of funding such as FLAG-ERA, for which three transnational calls were launched, designed in collaboration with the HBP scientific leadership. About a third of the projects emerged from the HBP Voucher calls which were launched twice by the HBP.

Therefore, some PPs focused on developing devices or methodologies (physical or digital), other addressed fundamental questions at different scales, or using different models (living or digitized, from different species), and/or for different brain disorders (see Fig. 1*B*).

### EBRAINS, a distributed research infrastructure for brain research, developed by the HBP

The goal of HBP was to develop tools and services for brain and brain-inspired research, and more specifically to create a major public data resource platform and offer access to large-scale simulations. In this framework, the release of six HBP Information and Communication Technology (ICT) platforms that were hosted by European partner institutions took place in 2016. These included the Neuroinformatics, Brain Simulation, Medical Informatics, High Performance Analytics and Computing, Neuromorphic Computing, and Neurorobotics platform. An urgent need to create links between the platforms led to the planning and development of the Joint HBP Platform in 2018. The aim of this Joint platform was to unify and coordinate the individual services developed in the HBP into one cloud-based super-structure available for different user groups and accessibility at different levels. To assure continuation and improvement of tools and services developed by HBP scientists after the end of the HBP, a new platform, named EBRAINS, was created in 2019. A corresponding legal entity was established and a web portal was officially released in 2019, thus allowing accessibility for the larger neuroscience community (Amunts et al., 2022). The EBRAINS RI has facilitated significant breakthroughs, which include the following key services (1) the Atlases service, allowing users to navigate through the mouse, rat, monkey and human brain and access different types of data; (2) the Data & Knowledge service, permitting access to large collections of datasets, models and software; (3) the Simulation service, providing powerful tools to conduct and validate simulation studies ranging from cellular to network to whole brain levels.

EBRAINS is a European distributed, digital, open, state-of-the-art research infrastructure that fosters collaborative brain science, providing tools and services that will assist scientists in their research, by collecting, analyzing, sharing, and integrating brain data and with special focus on performing modeling and simulation of brain functions (EBRAINS, 2023). EBRAINS has been included in the 2021 Roadmap of the European Strategy Forum on Research Infrastructures (ESFRI), having proved both scientific excellence and implementation rigor as a science infrastructure (ESFRI, November 2021).

EBRAINS services are made by and for scientists, following the principle of collaborative science and being constantly updated. Depending on the type of support users need and the type of data they aspire to use, they can access the services through the EBRAINS platform at different levels (i.e., Open services, Access-controlled services, and Services for selected users). The focus areas of EBRAINS cover and ensure equilibrium between neuroscience, brain health and brain-inspired technologies.

EBRAINS as a distributed RI is structured around National Nodes, which are groups of local European Institutions that will provide key EBRAINS services. These National Nodes are coordinated by the Central Hub (EBRAINS AISBL) located in Brussels, Belgium. This structure will support the evolution of the RI from a central European-funded project to a nationally funded RI. The aim of EBRAINS is to adopt a sustainable model that facilitates the continuous evolution and development of the RI, ensuring alignment with advancements in research and technology.

The HBP was the first EU-funded project coordinated by EBRAINS, following the coordination by the Ecole Polytechnique Fédérale de Lausanne (Switzerland). Besides, EBRAINS participated in multiple EU-funded projects (e.g., eBRAIN-Health, PHRASE, EHDS Pilot; visit https://www.ebrains.eu/projects for further information on these projects and others).

## An EBRAINS Workshop, a Platform for the HBP Partnering Projects

Especially since the establishment of EBRAINS, the PPs and their partners have been actively engaged and encouraged to pursue research that serves various objectives (1) adding novel capabilities to the EBRAINS RI; (2) using the RI to address issues in the field of neuroscience and technology; (3) developing novel computing and robotics technologies and applications; and (4) improving the understanding, diagnosis and treatment of brain disorders (Human Brain Project, 2021).

Researchers involved in the PPs have expressed positive feedback about the networking opportunities and improved visibility of their projects brought by the Partnering program. Paul Verschure and Julien Vezoli were appointed in 2020 as the PP Representatives, a role providing them access to the HBP Science and Infrastructure Board (SIB) through monthly meetings. This allowed them to express the clear need for PPs to have tangible and widespread scientific exchange. The final funding phase of the HBP (2020–2023) was the perfect timing to gather the PP members in a dedicated meeting to provide them with the opportunity to exchange knowledge and expertise and foster potential collaborations and fruitful discussions. Furthermore, PPs can be seen as a representative sample of the neuroscientific communities, to which it was important to give a voice.

Thus, the HBP PPs Meeting was organized in September 2022, with two main objectives: first, to give a platform to the PPs to showcase their achievements and extend their scientific network with other PPs and participants within and beyond the HBP environment. Second, to create new communities of interested scientists that could critically contribute, as users or developers of the EBRAINS services and tools and therefore to the evolution of EBRAINS as a sustainable infrastructure for the wider scientific community.

The PPs meeting was hosted by the annual Donders Cognitive, Brain & Technology Summer school (DCBT 2022) that promotes a systems-level understanding of the functional architecture of the brain and its possible emulation in artificial systems. The audience consisted of a neuroscience community that extended beyond the already far-reaching scope of the HBP community and 20 abstracts were presented.

The workshop program gathered PPs which showed interest in this opportunity and were available for the event, 23% of the all the HBP PPs were present, and the program was structured around focus areas of the presenting PPs. Four speakers were invited to present plenary sessions in line with the topic of the day:

For the Primate Brain Specifics session, Béchir Jarraya (member of the PP CORTICITY) presented the effects of deep brain stimulation (DBS) of the thalamus on restoring consciousness in anesthetized nonhuman primates (NHP). Experiments performed in NHP demonstrated that thalamic DBS specifically awakened monkeys from anesthesia by restoring anatomo-functional signatures of consciousness (Tasserie et al., 2022). Thalamic DBS restored the two main dimensions of consciousness, i.e., arousal and awareness, paving the way for therapeutic translation in patients with disorders of consciousness, such as in minimally conscious state patients (Demertzi et al., 2019).

For the Brain Circuits and Simulation session, Roshan Cools (HBP lead scientist) gave a lecture on the Chemistry of the Adaptive Mind from the angle of striatal dopamine, describing how dopamine (DA) is related to various aspects of human life and the clinical problem inherent to the huge interindividual variability in its effects (Cools et al., 2022). By combining psychopharmacology, fMRI, PET imaging and computational modeling of behavioral responses with a model of striatal DA, the project demonstrated that interindividual variability in response to catecholaminergic drug depends on individual differences in baseline DA synthesis capacity (Aarts et al., 2021; Rostami Kandroodi et al., 2021; van den Bosch et al., 2022). Future directions include importing this 100 subjects database into the EBRAINS Knowledge Graph for its use in a model simulating dopamine drug effects on human cognition.

For the Applied Brain Models session, Mehdi Khamassi (director at the Institute of Intelligent Systems and Robotics in Sorbonne University) presented his computational model of DA-based learning and exploration regulation. The model builds on the coordination of model-based (MB) and model-free (MF) reinforcement learning (RL), which was previously used to model rodent navigation (Dollé et al., 2018) and to drive a rat-like robot (Caluwaerts et al., 2012), taking advantage of both strategies. The model could predict individual differences in behavior obtained in rodents, based on the role of DA in regulating the exploration-exploitation trade-off and environmental variables (Cinotti et al., 2019; Lee et al., 2018), showing that different brain states use distinct models to adapt behavior.

Finally, Petra Ritter (co-lead of The Virtual Brain) presented in the closing plenary session, how multilevel health data can be used to enable complex simulations (Schirner et al., 2018). Behavioral and brain imaging results from the Human Connectome Project were used to run simulations and model cognitive function in 650 virtual brains, examining fast-but-faulty versus precise-but-slow decision-making. The results showed that the brain models based on subjects with better performance made on average more correct decisions but also took more time to take decisions (Schirner et al., 2023).

Following plenary sessions on the first 2 d, parallel sessions aimed to engage scientists and representatives from the EBRAINS Services into discussions on the use of the respective resources and on the outlook of the upcoming EBRAINS areas and services. It was important that the PP members were provided a comprehensive overview, update and future plans of the development of EBRAINS. This facilitated discussions related to EBRAINS’ benefit to current and potential users. By sharing information about existing tools and services, PP members were able to better grasp how scientific questions could be addressed using these resources.

The EBRAINS Atlases service was presented by Timo Dickscheid, focusing on the development of neuroinformatics solutions for online-accessible multimodal human brain atlas at cellular resolution, using deep learning methods for brain mapping and segmentation. The publicly accessible service includes software interfaces for accessing atlases, as well as tools and workflows for integrating data to the brain atlases and for their analysis. The multilevel human brain atlas has 390 subspaces and allows users to navigate via the 3D Interactive Atlas Viewer at nearly cellular level with the BigBrain model, with access to areal information about cells, fibers, receptors, and anatomic and functional connectivity. Future directions include increasing coverage of data features and datasets, integration of the multilevel connectome, and creation of a more complete NHP atlas.

The EBRAINS Data & Knowledge service was presented by Ingrid Reiten. This service has been curating data since 2018 with the goal of making it an available and useful service for the community. The Knowledge Graph combines metadata ingestion pipelines, human user input, and quality assurance processes to ensure consistent, high-quality data for contributors and users. Through this process, neuroscientists can make their data, models, and software available according to the FAIR (Findable, Accessible, Interoperable, and Reusable) principle (Wilkinson et al., 2016) and deliver data as open as possible and as closed as necessary. Various types of accessibility are offered, such as free access and access under embargo, controlled access, and restricted access. These services enable scientists to share, find, integrate, analyze, and simulate neuroscientific data.

The EBRAINS Simulation services were presented by Marmaduke Woodman. A special focus was given to the virtual brain (Schirner et al., 2022) although many other mature services are available for simulation and modeling (e.g., NEST, Elephant). These services enable testing mechanistic hypotheses and interpreting model driven data analysis. Several example user cases were presented including the TVB-NEST co-simulation software, an example of extensive association between tools accessible on the platform, bridging modeling to clinically relevant use (clinical trial started in 2019).

Lena Oden presented how EBRAINS provides Interactive Computing e-Infrastructure combining federated high-performance computing (HPC), Cloud & Storage infrastructure for neuroscience research. Access is free of charge and granted to users through an excellence-based peer-review procedure and submission of a proposal through the new online application system JARDS. Through the federated Fenix Authentication & Authorization Infrastructure, users have access to data repositories and scalable computing services. The advantage of using the service is that users have a single account to access all the services, and their proposal can include the application for resources at different HPC sites simultaneously.

Amaryllis Raouzaiou presented how the HBP Technical Coordination coordinates technical aspects of the transition to the EBRAINS RI. This work includes the development of guidelines and documentation for EBRAINS, involvement in co-designing of processes and collaboration with component owners, creating a roadmap for the services implementation and fostering collaboration with various partners within the HBP. Further steps involve incorporation of more use cases through user feedback for scientific workflow optimization and address of any software maintenance issues. The Technical Coordination is essential for the integration of services into the architecture of the EBRAINS RI.

In the rest of this paper, we present a summary of the work presented by the PPs during the 3-d meeting. We conclude by a summary of open discussions held with EBRAINS representatives, and discuss how to cooperate and contribute to the ongoing evolution of this digital platform at the service of brain researchers. This reflection takes into account the valuable lessons learned through the HBP PP program.

The PP meeting program is available online with direct links to most of the presentations and video recordings.

### Focus on primate brain specifics related partnering projects and EBRAINS services

The first session of the PP meeting shed light on investigations pursued in NHP research, in healthy and pathologic conditions but also through cross-species comparisons of brain structure and function with humans and/or rodents.

Nonhuman primate research is crucial to further our understanding of the link between structure and function of the primate-specific brain that include humans. Key aspects of human perception, cognition and behavior appear to depend on primate-specific specializations of the cerebral cortex, and this may explain why so many human neurologic and neuropsychiatric diseases are inadequately modeled in rodents (Izpisua Belmonte et al., 2015).

**Figure 2.**
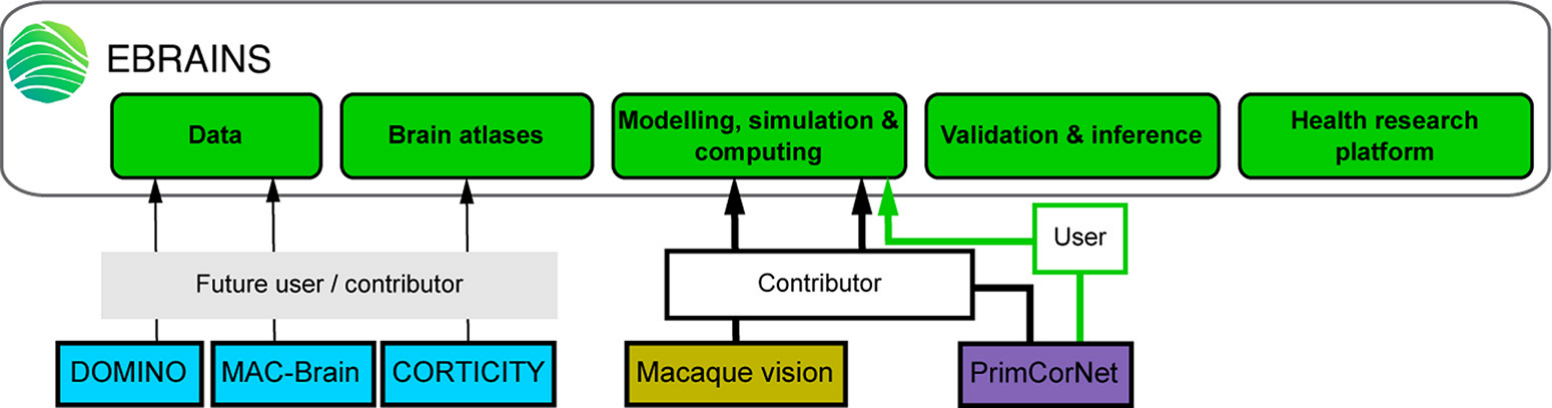
The Primate Brain Specifics HBP Partnering Projects categorized by HBP focus area (same color code as in Fig. 1*B*) and their relation to EBRAINS services (green boxes). Note: User = Individual already using EBRAINS tool/service for research; Contributor = Individual already contributing to the development of EBRAINS tool/service, enriching EBRAINS tools portfolio; Future user = Individual who plans to use the tool/service for research (statement made during the PP meeting and relevant surveys); Future contributor = Individual who plans to provide research data for the development of the EBRAINS tool/service (statement made during the PP meeting and relevant surveys).

In the HBP, several groups studied cross-species differences to enrich and validate models, datasets, or atlases over the course of the Project that are available at EBRAINS, and so did some of the Partnering Projects (see Fig. 2).

One example is the integration of the Multilevel Macaque Brain Atlas, MEBRAINS, in EBRAINS, that was presented by Wim Vanduffel. A preliminary version of the atlas, using a population-based template MRI with a 0.4 μm resolution, has been released and is designed to receive and integrate results from various domains of primate neuroscience research in a common space. For this to be achieved, interoperability is of paramount importance. The primate neuroscience community is formally invited to participate in the atlas implementation by feeding it with their results and it is ensured that they will receive support from the data curation team and also for development of new user services. NHPs have been and remain an essential model system to further understanding and modeling of primate-specific brain function, and the NHP Atlas holds great promise for centralizing the wide diversity of NHP research performed at multiple levels and scales.

Another example of knowledge and tools available in EBRAINS is related to the PRIMCORNET. This project, presented by Bjørg Kilavik and Sacha van Albada, combined experimental (Gregoriou et al., 2012; Confais et al., 2020) and modeling approaches to provide novel NHP electrophysiological datasets, validation tools and a multiarea cortical model available through EBRAINS (Schmidt et al., 2018), paving the way to neurorobotics simulations.

Macaque vision, presented by Simo Vanni, combined biological and computational approaches, notably EBRAINS tools (e.g., the CxSystem2 software, accessible through the Knowledge Graph) for data-driven brain models and simulation, to create a physiologically plausible working model of early cortical vision in primates (Hokkanen et al., 2019; Vanni et al., 2020; Garnier Artiñano et al., 2023). This project furthermore aims to develop a V1 model using the Grossberg ART model (Grossberg, 2021) as a scaffold.

DOMINO, presented by Conrado Bosman, investigated how impairments in multisensory integration are key characteristics of autism spectrum disorders. This project used a multispecies (rodent, human) multiscale approach (Arbab et al., 2018; Oude Lohuis et al., 2022) to develop a computational model of multisensory integration and cross-modal plasticity that will be integrated into the EBRAINS platform.

MAC-Brain, presented by Elisa Santandrea, focused on understanding how distinct attentional control signals interact in the primate brain to build a unique spatial priority map for guiding the deployment of visual selective attention, at the service of both target selection and distractor suppression. Human EEG and fMRI data testing the influence of multiple attentional control signals have been acquired using standardized behavioral protocols (Beffara et al., 2022; Dolci et al., 2022a,b), and will be complemented by NHP recordings with the same tasks; all these data will be shared through EBRAINS.

CORTICITY, presented by Julien Vezoli, exploited invasive techniques in mouse and NHP to extract empirical data and identify fundamental principles on cortical organization (Gămănuţ et al., 2018; Froudist-Walsh et al., 2021; Vezoli et al., 2021; D’Souza et al., 2022), both essential for the development of whole-brain models and large-scale simulations (Deco et al., 2021; Hahn et al., 2021; López-González et al., 2021; Signorelli et al., 2021). Comparing simulations to noninvasive recordings in NHP and humans identified principles of the primate cognitive architecture supporting different states of consciousness (Ponce-Alvarez et al., 2022).

While these projects represent only a fraction of PPs that have contributed to significant progress in the understanding of the specificity of the primate brain circuits and function, they illustrate beautifully how PPs contributed to the enrichment and development of EBRAINS tools and services often through direct collaborations with the HBP (Hahn et al., 2021; López-González et al., 2021; Vezoli et al., 2021).

### Focus on brain circuits and simulation related partnering projects and EBRAINS services

**Figure 3.**
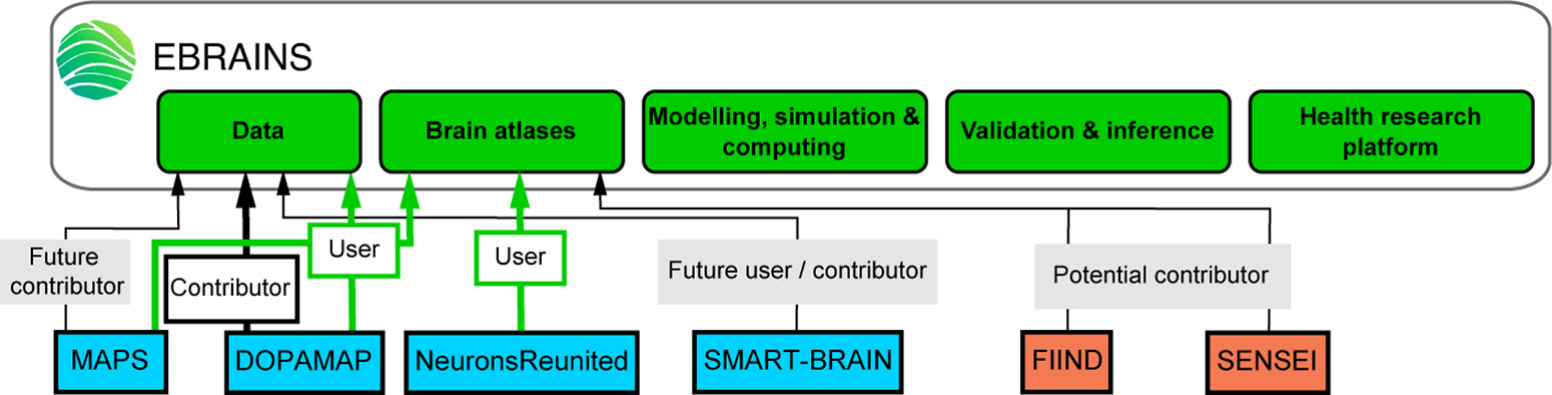
The Brain Circuits and Simulation HBP Partnering Projects categorized by HBP focus area (same color code as in Fig. 1*B*) and their relation to EBRAINS services (green boxes). Note: User = Individual already using EBRAINS tool/service for research; Contributor = Individual already contributing to the development of EBRAINS tool/service, enriching EBRAINS tools portfolio; Future user = Individual who plans to use the tool/service for research (statement made during the PP meeting and relevant surveys); Future contributor = Individual who plans to provide research data for the development of the EBRAINS tool/service (statement made during the PP meeting and relevant surveys); Potential contributor = Individual who considers to contribute to the tool/service but has no immediate plans or resources to contribute.

Exploring and mapping brain circuits is critical to build efficient computational brain models. Most of the PPs involved in this field exploited tools offered by the EBRAINS RI that also served for running simulations to test working hypotheses. They also supported development of the EBRAINS tools and services through their work (see Fig. 3).

During a dedicated session, HBP PPs presented their progress on developing methodologies and tools, and their acquisition of empirical data necessary for the building of digital brain circuits.

MAPS, presented by Eleonora Centofante, investigated the changes in cellular activity (c-Fos) patterns induced by spatial learning in Rodent hippocampus (Mastrorilli et al., 2022). To better understand brain dynamics during spatial learning, they developed a network model using the Fenix RI showing that training procedure can be decoded from hippocampal activity pattern. They plan to share their data with the EBRAINS platform.

DOPAMAP, presented by Jee Hyun Kim, produced maps of dopamine receptors 1 and 2 in the developing mouse forebrain and provided a comprehensive collection of high-resolution microscopic images, registered to the Allen Mouse Brain Common Coordinate Framework and shared via the EBRAINS Knowledge Graph (see, e.g., dopamine 1 receptor distribution). Data are available for download or direct inspection through an interactive web-microscopy viewer. The DOPAMAP collection (Bjerke et al., 2022) is an important resource for researchers, and also explores the use of brain atlases for the developing mouse brain.

FIIND, presented by Roberto Toro, aims to create a detailed description of the development of the ferret brain. This project is an open, collaborative effort to generate the first integrated atlas of ferret brain development and is the basis for an open platform for creating collaborative multimodal, multiscale, brain development atlases. The web platform allows researchers to access and visualize the data and create collaborative, human-curated, 3D segmentations of brain structures.

SENSEI, presented by Nicola Vanello, developed new methodologies and tools for neural tissue processing, imaging, and neuron segmentation at different levels, ranging from neuronal shape to finer structures such as dendritic spines based on topological and image intensity information. In addition, within SENSEI a new fast, super resolution-compatible imaging system based on a line-scan confocal imaging device (TriScan) was developed. The next steps foresee testing segmentation algorithms (Callara et al., 2020) on samples acquired with different modalities, tissue labeling and processing techniques, including samples from humans.

NeuronsReunited, presented by Nestor Timonidis, developed tools for accelerating the reconstruction and spatial registration of small subset of carefully selected thalamic neurons, as well as for mapping out and visualizing their axonal projection patterns alongside thousands of publicly available neurons in a 3D reference space. Using these data and the NEST simulation environment (Potjans and Diesmann, 2014), they developed a model for simulating the interactions between the lower order thalamic nuclei and the somatosensory barrel cortex. Future directions include incorporating multicompartmental neuron simulations using NEURON (Hines and Carnevale, 2001) to study propagation delays in the above model.

SMART-BRAIN, presented by Jonathan Mapelli and Raf Van de Plas, is developing advanced morphologic reconstruction of human brain tissue through multimodal fusion of multiscale optical imaging technologies. The cross modal fusion model allows for prediction of one modality’s signal on the basis of another modality in areas of the brain where the first modality cannot be acquired and only the second modality is measured (Van de Plas et al., 2015). The objective is to import cross modal models and their predictions into EBRAINS.

Building digital brain circuits for realistic brain simulations based on accumulated empirical observations integrated into 3D reference atlases (Leergaard and Bjaalie, 2022) is one core aspect of EBRAINS services, in which PPs have played an important role (Bjerke et al., 2022).

### Focus on applied brain models and Responsible Research and Innovation (RRI), related Partnering Projects, and EBRAINS services

**Figure 4.**
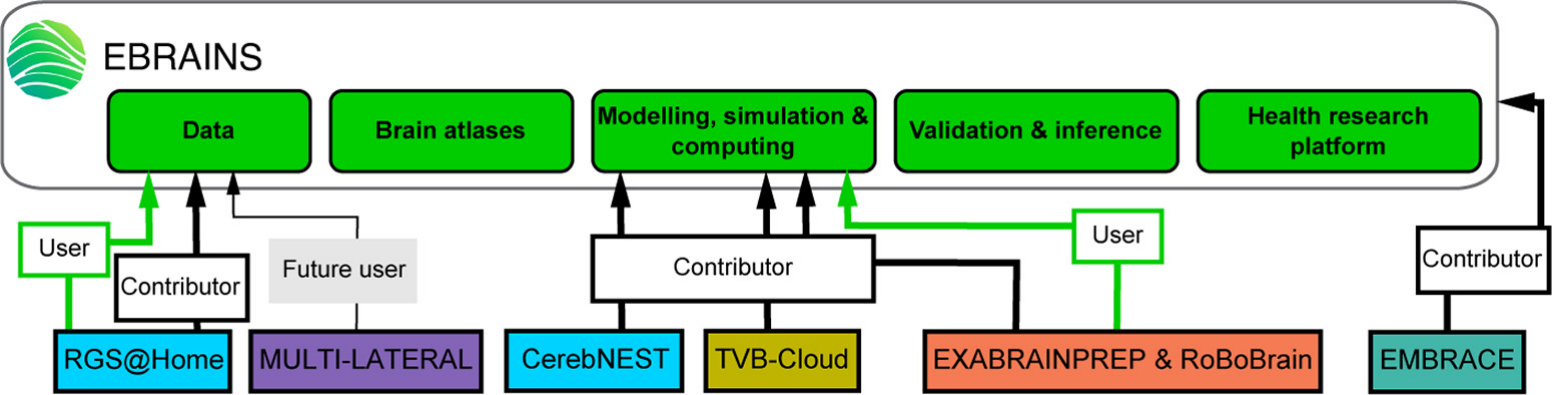
Applied Brain Models and Responsible Research and Innovation (RRI) HBP Partnering Projects categorized by HBP focus area (same color code as in Fig. 1*B*) and relation to EBRAINS services (green boxes). Note: User = Individual already using EBRAINS tool/service for research; Contributor = Individual already contributing to the development of EBRAINS tool/service, enriching EBRAINS tools portfolio; Future user = Individual who plans to use the tool/service for research (statement made during the PP meeting and relevant surveys).

One of the major goals of brain computational models, beyond a mechanistic understanding of how brain microstructure and connectivity leads to behavior and function, is to apply these models to simulate therapeutic interventions and to embody them into robotic applications for improving quality of life of human patients. Therefore, a session was organized on PPs aiming toward the development of models with different levels of application, ranging from digital reconstruction based on cross-modal fusion to personalized medicine based on a digital twin. The relation of these PPs with the HBP and EBRAINS is illustrated in Figure 4.

CerebNEST, presented by Alessandra M. Trapani, is a bioinspired multiscale modeling of the cerebellar network (Casellato et al., 2014; Geminiani et al., 2019), developed using the NEST-simulator in EBRAINS. Recent developments include robotic embodiment and a diffusive plasticity mechanism, making it more realistic and detailed (Trapani et al., 2021; Antonietti et al., 2022).

EXABRAINPREP & RoBoBrain, presented by Carlos Enrique Gutierrez, target two different aspects of computational models of the brain: technical and scientific. Different models of the brain and large-scale simulations were built and ran using NEST but were also deployed on Japan’s new flagship supercomputer Fugaku (Feldotto et al., 2022; Gutierrez et al., 2022). Effort is now directed at scaling these models to marmoset, macaque, and human brain sizes. Partnering with the Neurorobotic Platform (NRP) and RoBoBrain includes improving and validating large-scale models by adding constraints from musculoskeletal systems in the NRP, implementing learning tasks such as reinforcement learning, and experimenting in the NRP.

Some PPs were also focusing on more clinical and applied research.

MULTI-LATERAL, presented by Clyde Francks, is a multilevel integrative analysis of brain lateralization for language, focusing on brain asymmetry and its potential link to a series of psychiatric disorders (Postema et al., 2019; Sha et al., 2021a,b). Multivariate genome-wide association of 42 regional brain asymmetries in over 34,000 adults found 21 genomic loci associated with variation in adult brain structural asymmetry, and evidence for a genetic overlap with autism and schizophrenia (Sha et al., 2021a,b).

RGS@HOME, presented by Paul Verschure, is a research initiative where the goal is to provide personalized 24/7 home care for neurorehabilitation using ICT-based tools. This project seeks to conduct more applied research to bring this technology from laboratory to home, with the aim of improving the quality of life for people with neurologic conditions (Mura et al., 2022).

The Virtual Brain Cloud (TVB-Cloud), presented by Petra Ritter, is a digital “brain twin” that offers GDPR-compliant solutions for accessing personal health data in an open infrastructure (Schirner et al., 2022). TVB-Cloud includes six services integrated in EBRAINS, recent innovations include multiscale simulation, digital drugs, *in silico* deep brain stimulation, augmenting diagnosis and prediction, embodied virtual brain, and cognitive virtual brain. These services allow connecting data sources to users and enable running complex simulations of multiscale digital twins in a lawful setting, prioritizing security and privacy.

The HBP also had a strong focus on ethics and society research, including Responsible and Research Innovation (RRI) activities. One of the PPs was working with members of the HBP: EMBRACE. EMBRACE, presented by Mayen Cunden, is developing services to integrate principles of future and emerging technologies research, using for example applied brain models and Artificial Intelligence. Ethical issues can arise from the intersection of neuroscience and ICT, and RRI needs to address them. What approach should EBRAINS and other neuroscience research infrastructures take in the implementation of responsible practices? A specific Ethics Taskforce has been formed in EBRAINS to ensure these practices are followed in the future, in the timeframe between the meeting and the writing of this report.

Through the development of applied brain models, PPs have significantly contributed to the HBP objective for the development of personalized network models (Schirner et al., 2022) and relevant clinical applications to better inform personalized medicine (Jirsa et al., 2023).

## Next Steps. Outlook

In the 10-year project, the HBP has accepted 76 Partnering Project applications. The EBRAINS workshop held in September 2022 provided an opportunity to gain insights into the progress of some PPs in their respective scientific domains. Additionally, the workshop facilitated discussions about the present and future development of the EBRAINS Research Infrastructure.

Over the years, clear links between the HBP and its PPs have been difficult to map, as collaboration with HBP members was not mandatory (although in many instances members of these projects were also members of the HBP), but also because the PPs had no obligations to report to the HBP partnering about their progress. However, one of the major advantages of the Partnering framework of the HBP lied in the networking opportunities, exemplified by this EBRAINS workshop, or in the yearly HBP summits.

Yet, beyond bringing significant progress in the fundamental understanding of the relationship between brain circuits, function and behavior (Gregoriou et al., 2012; Demertzi et al., 2019; Froudist-Walsh et al., 2021; Deco et al., 2021; Schirner et al., 2023), PPs have directly contributed to the development of whole-brain models and digital platforms exploiting latest Artificial Intelligence and HPC technologies to create next-generation robots such as haptic telerobot hand (https://www.shadowrobot.com/). They also proposed personalized medicine services ranging from prospective screening and therapeutic intervention to clinical outcome monitoring, which have been substantiated by clinical trials (e.g., NCT05159661, NCT04620707, NCT03643016, NCT02603640), innovative medicines initiatives (e.g., https://www.radar-cns.org/, https://virtualbraincloud-2020.eu/) and European reference networks linking health care providers for multicenter large cohort studies (e.g., https://epi-care.eu/, https://www.ai-mind.eu/; Giugni et al., 2021).

In the final phase of the HBP, engagement activities were increased to enhance community awareness about services provided by EBRAINS and to potentially expand the community and attract future users, focusing on three primary profiles: users, co-developers, and future contributors.

Through the discussions held during the EBRAINS workshop, organized by the representatives of the HBP PPs, it became evident that the scientific community beyond the HBP and the Partnering environment not only has the potential to contribute but is a crucial player for the future development of EBRAINS. Their involvement is essential in promoting the progress of brain science through the infrastructure.As emphasized by Jan Bjaalie and Steven Vermeulen, collaborative projects are now needed to build a strong infrastructure.

The workshop concluded with Steven Vermeulen (EBRAINS), Paul Verschure (Donders Institute for Brain, Cognition and Behaviour), and Julien Vezoli (Ernst Strügmann Institute for Neuroscience in cooperation with the Max Planck Society) all emphasizing the importance of the Partnering Projects for the future development of EBRAINS. They envision EBRAINS to be a platform for collaboration, providing a common language, methods, and concepts to advance understanding of the brain. Finally, they discussed the need for EBRAINS to support projects from fundamental research to more applied levels for patients’ benefit. Julien Vezoli noted that Partnering Projects are not only contributing but, are also critical for the future development of EBRAINS. Paul Verschure believes that EBRAINS can be a fuel to bring communities together, creating a common language and methods, and advance understanding of the brain. Steven Vermeulen sees the start of a new normal with EBRAINS, supporting projects that need more than just services and tools but also a collaborative platform that we would build together.

EBRAINS supports different types of projects and needs, ranging from individual researchers to research organizations (Vermeulen, 2022), offering advantages to researchers, research groups, consortia, research organizations and is continuously looking for new projects to continue development of its services. The future orientation of EBRAINS projects will be driven by a thematic focus on specific research questions (including fundamental research and applications toward health), the service orientation toward building tools, services and data resources, and the ambition of providing brain-inspired technologies (Amunts et al., 2022). EBRAINS is currently participating in a series of consortia that are funded and that are directed toward brain health. Additionally, EBRAINS members and its central hub have been actively engaged in identifying communities and submitting applications to international funding calls focused on brain health. Some members of the HBP PPs are part of this ecosystem. The European-funded PHRASE project is one of them.

Ideally, in the future, funders should prioritize supporting the utilization of infrastructure such as EBRAINS to harness the existing knowledge and tools that have been developed. Such Research Infrastructures, and EBRAINS specifically, shall continue to promote the integration of research outcomes back into its platforms to ensure a virtuous circle between users, developers, and providers to ensure constructive accumulation of empirical knowledge based on a common ground and to emulate new discoveries based on maintaining free access to the large scientific community. The mechanism of association between an RI and projects should clearly state the expectations from both the RIs and the researchers. RIs should actively track and monitor the usage of their infrastructure by various projects. They should promote the integration of project outcomes into the RI to ensure a virtuous circle of continuous improvement and advancement.

## Synthesis

Reviewing Editor: Niraj Desai, National Institute of Neurological Disorders and Stroke Decisions are customarily a result of the Reviewing Editor and the peer reviewers coming together and discussing their recommendations until a consensus is reached. When revisions are invited, a fact-based synthesis statement explaining their decision and outlining what is needed to prepare a revision will be listed below. The following reviewer(s) agreed to reveal their identity: NONE.

Thank you for submitting this manuscript to eNeuro. It has now been read by two expert reviewers and by me. We all agree that the results and ongoing work of the European Human Brain Project (HBP) are of broad interest to the neuroscience community and merit wide dissemination. The manuscript is clearly and well written but is also, to be honest, a fairly dull read. It is a listing of projects and resources, with links and brief descriptions, but without a unifying thematic framework. As the second reviewer notes below, the manuscript would generate much greater interest and have a larger impact if it included some attempt to synthesize the work, to give readers some idea of how the disparate parts form a whole, to indicate how the work so far supports the long-range goals of the HBP. This is a challenging writing task, but it principally involves recrafting the introduction and we think it is worth the effort because doing so would increase the value and the impact of this publication in a substantial way.

I have appended the reviewer comments below.

REVIEWER 1

I appreciate the clear and succinct summaries (including links to data/source) of each of the major sessions. The manuscript provides a historical and future outlook of the HBP, as well as a good resource to the audience of the major achievements.

REVIEWER 2

This review covers progress made regarding partnering projects related to the European Human Brain Project (HBP), specifically centred on reporting on the various outcomes of a recent meeting that was held in September 2022. The review is mainly organised around listing the various online forums that have been created with a view to creating a level of synergy among several related research endeavours.

While potentially useful as a resource, this presents as a fairly dull read and I wonder if this overview of an exciting body of research could have been showcased in a more engaging way. For a group of investigators interested in how the brain works, there seems to be a surprising lack of imagination in how to group these myriad resources together under a common, logical structure. This is exemplified by the one figure that has been provided (Figure 1): a list of 6 HPB promises, and a corresponding matched list of statements on how these promises have been fulfilled. HBP ends in 2023, so this seems to be called for, but this figure is not helpful. Similarly, throughout the review are listed a range of online forums or resources (PRIMCORNET...DOMINO...CerebNEST, etc) with brief descriptions and associated references. One could question the value of publishing a review with these resources presented this way, without any overarching logic or visual guide.

While I was certainly not expecting an exposition on some of the interesting neuroscience that came out of the HBP, I suspect the average reader might appreciate more of a synthesis of how these partnerships relate to one another, and how they might be achieving something novel beyond just being online manifestations of existing research programs. Clearly there must be a visually intuitive way of grouping and linking these various forums so it becomes clearer how they contribute to the overall goals of the HBP? Some involve resources such as brain atlases, others are computational models to which users might add data, others are involve data repositories and services. For this to be a useful read for other scientists beyond the audience that attended the 2022 meeting, I might recommend coming up with some schemas or diagrams that explain how these myriad platforms are connected and related under the larger EBRAINS umbrella. This might be a flowchart or network diagram, but the value for the reader would be to immediately recognise the different groupings related to particular research needs, which would then allow them to find them again in the text and be able to extract more meaning in that way. The current style with a list of names and one largely uninformative figure does not help make this an easily digestable review.

Beyond this organisational weakness, I suspect the average reader might also appreciate an attempt towards some level of synthesis where it might be made clearer how these partnership programs created a new way of thinking or doing research that was not possible before. It would have been interesting to conclude with such observations, including specific examples, rather than re-iterating the importance of creating platforms for collaboration. Of course this is important. But perhaps outline a few examples of things that were discovered or research directions that were enabled that might not have happened without said platforms? In other words, I suspect the average reader will be craving for at least a bit of cutting edge neuroscience at the end of this read, after having understood that this review is mainly a lists of online groupings and resources that have emerged as a consequence of the HBP. It would be unfortunate to miss this opportunity to provide something more here.

There are a few minor grammatical corrections:

Line 109: ...in specific EU funded that will... (funded what?)

Line 150: A stray ‘_’

Line 155: A stray ‘do’

Line 350: A stray Elephant. Or perhaps explain or provide a reference?

Figure 1: Top right box: ‘are build’ should be ‘are built’

Figure 1: Bottom right box: last sentence, an ‘and’ seems to be missing.
